# Genome-Wide Association Study of Major Agronomic Traits Related to Domestication in Peanut

**DOI:** 10.3389/fpls.2017.01611

**Published:** 2017-09-26

**Authors:** Xingguo Zhang, Jianhang Zhang, Xiaoyan He, Yun Wang, Xingli Ma, Dongmei Yin

**Affiliations:** College of Agronomy, Henan Agricultural University Zhengzhou, China

**Keywords:** peanut, domestication, genome-wide association studies, selective sweeps, single-nucleotide polymorphisms (SNPs)

## Abstract

Peanut (*Arachis hypogaea*) consists of two subspecies, *hypogaea* and *fastigiata*, and has been cultivated worldwide for hundreds of years. Here, 158 peanut accessions were selected to dissect the molecular footprint of agronomic traits related to domestication using specific-locus amplified fragment sequencing (SLAF-seq method). Then, a total of 17,338 high-quality single nucleotide polymorphisms (SNPs) in the whole peanut genome were revealed. Eleven agronomic traits in 158 peanut accessions were subsequently analyzed using genome-wide association studies (GWAS). Candidate genes responsible for corresponding traits were then analyzed in genomic regions surrounding the peak SNPs, and 1,429 genes were found within 200 kb windows centerd on GWAS-identified peak SNPs related to domestication. Highly differentiated genomic regions were observed between *hypogaea* and *fastigiata* accessions using *F*_*ST*_ values and sequence diversity (π) ratios. Among the 1,429 genes, 662 were located on chromosome A3, suggesting the presence of major selective sweeps caused by artificial selection during long domestication. These findings provide a promising insight into the complicated genetic architecture of domestication-related traits in peanut, and reveal whole-genome SNP markers of beneficial candidate genes for marker-assisted selection (MAS) in future breeding programs.

## Introduction

Peanut, also known as groundnut (*Arachis hypogaea* L.), is one of the most important edible oil crops in the world. Cultivated peanut is an allotetraploid (AABB, 2n = 40), harboring homoeologous A and B genomes putatively derived from the natural hybridization of two wild diploid species, *A. duranensis* (AA, 2n = 20) and *A. ipaensis* (BB, 2n = 20) (Seijo et al., 2007; Moretzsohn et al., 2012). Based on the presence or absence of floral axes on the main stem, cultivated peanut is classified into two subspecies: *hypogaea* and *fastigiata*. Subspecies *hypogaea* is generally described as having a prostrate growth habit with no floral axes on the main stem, while in subspecies *fastigiata* flowers arise on leaf axils on branches as well as the main stem (Krapovickas and Gregory, 1994).

As the only cultivated species of *Arachis*, peanut has been grown for hundreds of years in more than 100 countries worldwide (Huang et al., 2012). The evolution of *Arachis* is therefore closely related to the domestication of cultivated peanut, with a vast number of morphological forms having evolved under cultivation. For example, selection of a more upright growth habit and shorter branches, which allow easier harvesting and increased seed size, has also resulted in a decrease in resistance to a number of important pathogens (Stalker and Simpson, 1995; Stalker et al., 2013). Domestication-related quantitative trait loci (QTLs) associated with agronomic traits and resistance have already been mapped; however, the utilization of potential alleles has been relatively limited because of the lack of appropriate molecular tools for analysis of these traits in cultivated peanut (Burow et al., 2001; Chu et al., 2011; Ravi et al., 2011; Fonceka et al., 2012; Tseng et al., 2016; Zhou et al., 2016).

With the development of high throughout sequencing technologies, whole-genome sequencing (WGS) has become much more straightforward, allowing analysis of the impact of domestication on genomic variation. Specific-locus amplified fragment sequencing (SLAF-seq) is an efficient method of large-scale single nucleotide polymorphism (SNP) identification and genotyping using high-throughput sequencing, with many advantages such as lower costs and reduced genome complexity (Mamanova et al., 2010; Sun et al., 2013). So far, this new method has been successfully used to address fundamental questions regarding soybean domestication (Han et al., 2016).

Genome-wide association studies (GWAS) have also been used to determine the genetic basis of traits underlying domestication in a wide range of organisms (Lin et al., 2014; Han et al., 2016). However, information on peanut domestication remains limited, largely due to the relatively large size (~2.8 Gb) and complexity of the tetraploid peanut genome (Bertioli et al., 2014). However, in 2014, genomes of the two wild progenitors of cultivated peanut were released by the International Peanut Genome Initiative (IPGI), benefiting studies of agronomic traits related to domestication (Bertioli et al., 2016).

In the present study, high quality SNPs distributed throughout the peanut genome were mined using SLAF-seq of 158 peanut accessions. GWAS was subsequently conducted to identify the genetic architecture of 11 major agronomic traits related to domestication. The results present the first comprehensive view of genome-wide sequence variation in a diverse group of peanut accessions. Moreover, the SNPs and candidate genes related to major agronomic traits will help accelerate peanut breeding programs.

## Materials and methods

### Plant materials and trait analyses

A total of 158 peanut (*A. hypogeae* L.) accessions were examined in the present study, including 36 *hypogaea* type (group I) with no floral axes on the main stem, and 122 *fastigiata* type (group II) with flowers growing on both branch and the main stem (Table S1). These accessions were elite cultivars collected from different provinces of China, and some of them were greatly produced in special areas.

Seeds from a single plant of each of the 158 accessions were grown in a randomized complete block design with three replications. Four plants from each replicate were then selected to investigate the following 11 agronomic traits: height of the main stem, total number of branches, branch type, leaf color, pod length, pod width, seed length, seed width, 10-pod weight, 10-seed weight and seed coat color.

### SLAF sample preparation and sequencing

Genomic DNA was isolated from fresh leaves of a single plant per accession, and analyzed using SLAF-seq (Sun et al., 2013). To obtain >200,000 SLAF tags per genome, evenly distributed in unique genomic regions, different restriction enzyme combinations were tested using *in silico* digestion prediction. Two restriction enzymes (*Rsa*I and *Hae*III) were selected based on uniqueness and uniformity of simulated fragment alignments to the reference genome sequence of two diploids, *A. ipaensis* (ftp://ftp.ncbi.nlm.nih.gov/genomes/all/GCA_000816755.1_Araip1.0, gene model is prefixed by Araip) and *A. duranensis* (ftp://ftp.ncbi.nlm.nih.gov/genomes/all/GCA_000817695.1_Aradu1.0, gene model is prefixed by Aradu). Different length fragments of genomic DNA after digestion were then simulated *in silico*. *Oryza sativa indica* (http://rapdb.dna.affrc.go.jp/) was selected as the control genome to test the accuracy of the restriction enzyme digestion protocol using SOAP software (Li R. et al., 2009).

A total of 10-ug of genomic DNA from each accession was used for the restriction reaction and subsequent restriction-ligation reactions, including the addition of A to the 3′ end and ligation with the Dual-index adapter. PCR was performed with the restriction-ligation samples (diluted) then the PCR products were purified with a Quick Spin column (Qiagen, Hilden, Germany) and electrophoresed on 2% (w/v) agarose gel. Fragments with expected lengths were isolated using a Gel Extraction Kit (Qiagen) and diluted for sequencing. Fragments of 314–344 bp were isolated for use as SLAF tags.

### SNP calling

All reads were processed for quality control and filtered using Seqtk (https://github.com/lh3/seqtk). High quality paired-end reads were mapped onto the reference genome (*A. ipaensis* and *A. duranensis*) using the Burrows-Wheeler Aligner (BWA) (Li and Durbin, 2009). Realigner Target Creator and InDel-Realigner in GATK (McKenna et al., 2010) were used to realign InDels, and Unified Genotyper was used to call genotypes across the 158 accessions using the default parameters. Sequencing depths of each sample were calculated using the “Depth of Coverage” module of GATK. Single SNP markers were confirmed using GATK (McKenna et al., 2010) and SAMtools (Li H. et al., 2009). Given the allotetraploid nature of the peanut genome, the genotyping errors caused by partial homologous alignment were resolved by comparing the sequencing depth first, then filtered those SNPs with integrity (genotyped rate) and minor allele frequencyand (MAF). The exceptionally high homologous regions were not under special analyses, because there were not too much SNPs were found in these regions.

### Population structure analysis and phylogenetic tree construction

Population structure was calculated using ADMIXTURE software (Alexander et al., 2009). The number of genetic clusters (K) was predefined as 1–10 to explore the population structure of the tested accessions. This analysis provided maximum likelihood estimates of the proportion of each sample derived from each of the K populations. SNPs were then used to calculate genetic distances among the 158 accessions, and phylogenetic trees were constructed using the neighbor-joining method in MEGA5 (Tamura et al., 2011). Principal component analysis (PCA) was performed using GAPIT software (Lipka et al., 2012).

### GWAS of agronomic traits

High-integrity SNPs from the tested peanut accessions were used in association analyses using the general linear model (GLM) and compressed mixed linear model (MLM) with TASSEL software (Bradbury et al., 2007). The following formula was used:

Y=Xa+Qb+Ku+e

where Q is the population structure derived from ADMIXTURE software (Alexander et al., 2009), K is the relationship between samples obtained from SPAGeDi (Hardy and Vekemans, 2002), using Q in GLM and Q + K in MLM. X represents the genotype and Y the phenotype, allowing associated values of each SNP to be calculated. A value of <0.01 was used as the threshold to determine the existence of a significant association. Gene predictions were annotated according to the method used in Zhang et al. (2015). Candidate genes associated with each trait located within a 100-kb region upstream or downstream of peak SNPs, because the size of the larger linkage disequilibrium (LD) Block is mostly distributed around 200 kb when the whole genome was analyzed for LD Block. The *r*^2^ value (Marker_Rsq, is the marginal R-squared for the marker) was used to explain the phenotypic variation of each marker. It was calculated as SS Marker (after fitting all other model terms) / SS Total, where SS stands for sum of squares (https://bitbucket.org/tasseladmin/tassel-5-source/wiki/UserManual/GLM/GLM).

### Population differentiation (*F_ST_*) and putative selective sweeps

The divergence index, *F*-statistics (*F*_*ST*_), is a measure of population differentiation or genetic distance based on genetic polymorphism data (Hudson et al., 1992). To determine potentially differentiated regions, *F*_ST_ estimations and sequence diversity (π) ratios were evaluated using a 100-kb sliding window with 10-kb steps (Lam et al., 2010). Highly differentiated genomic regions with a significant *F*_ST_ value (*p* = 5%) and the top 5% of π ratios were defined as potential selective sweeps (Li et al., 2013).

## Results

### Phenotypic variation among peanut accessions

**Figure 1:**
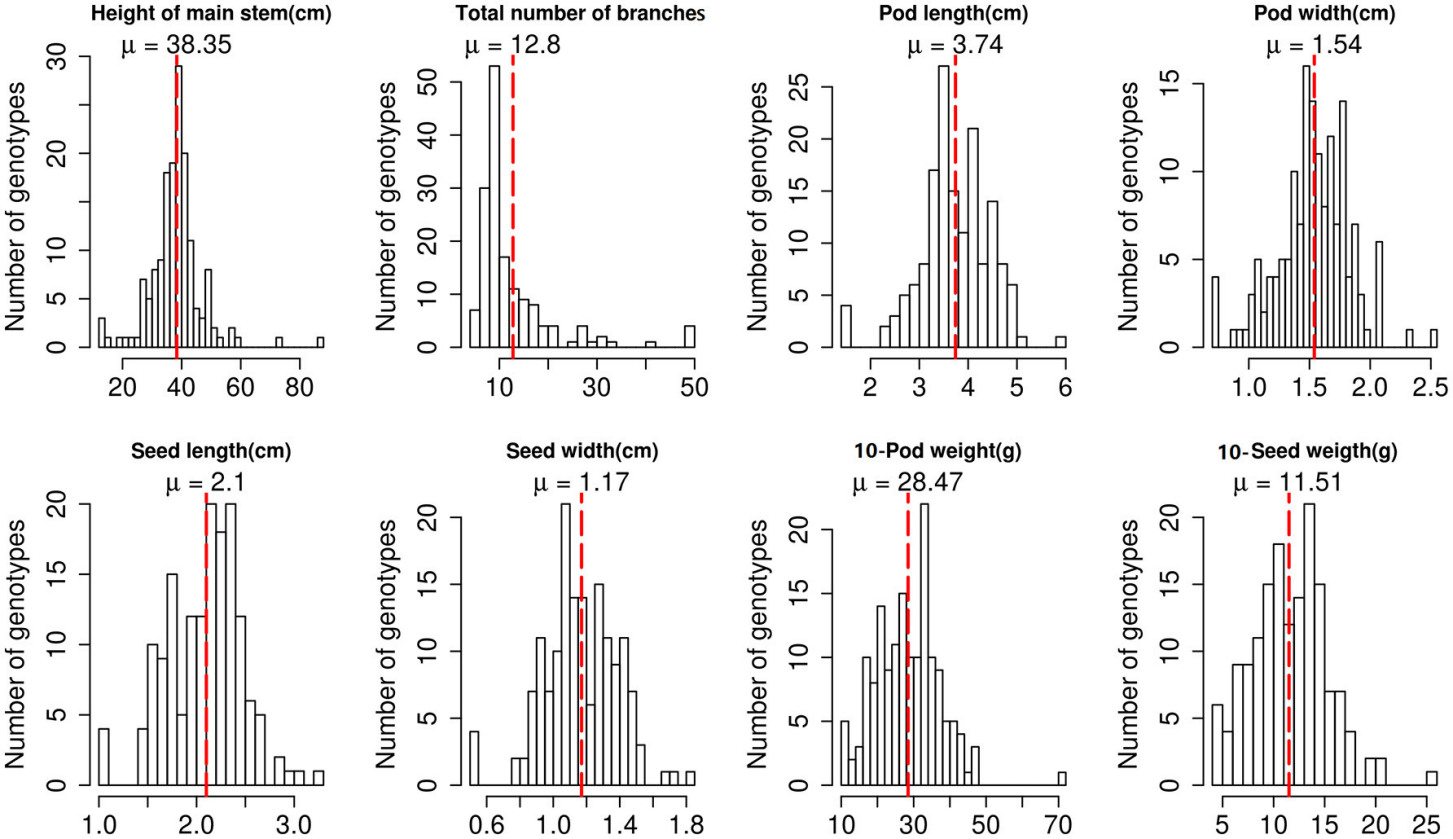
Phenotypic variation of eight traits among 158 peanut accessions.

Phenotypic evaluation revealed a broad range of variation among the 158 peanut accessions (Figure 1, Table S1). The descriptive statistics of phenotypic variation of eight traits were listed in Table S2. Height of main stem ranged from 12 to 87.33 cm, with an average of 38.35 cm. Total number of branches also varied with an average of 12.8. All accessions showed continuous distribution of the three pod-related and three seed-related traits, with a coefficient of variation (CV) of 18.63% for seed length and 66.98% for the total number of branches, suggesting a quantitative inheritance pattern. The remaining three traits, branch type, leaf color and seed coat color, were evaluated as quality variation.

### SLAF-Tags and SNP data

A total of 369,725 high quality SLAFs evenly distributed on 10 A and 10 B chromosomes were obtained from 397.19 M paired-end reads after sequence alignment with the reference genome (*A. duranensis* and *A. ipaensis*). Sequencing depths ranged from 32.42 to 4.94 X, with an average of 8.06 X. Polymorphic SLAFs defined by both GATK and SAMtools were recorded as reliable SNPs, resulting in a total of 268,889 SNP markers among the 158 accessions. After filtering SNPs located on scaffolds, 17,338 high quality SNPs with an MAF > 0.05 and integrity >0.5 were selected for further analyses (Table 1, Figure S1).

**Table 1:** Distribution of 17,338 SNPs in 20 chromosomes identified in 158 peanut accessions.

**Chr**	**Chr length(Mb)**	**Number of SNPs**	**Number of genes**	**Average number of SNPs per Mb**	**Average number of genes per Mb**	***r*^*2*^ of chr LD**
A01	106.02	725	3493	7	33	0.177
A02	92.63	1059	3129	11	34	0.356
A03	133.14	970	4929	7	37	0.071
A04	121.18	749	3833	6	32	0.185
A05	108.28	793	3868	7	36	0.166
A06	110.73	862	3712	8	34	0.149
A07	77.95	482	2850	6	37	0.161
A08	48.94	314	2940	6	60	0.151
A09	119.00	887	3694	7	31	0.173
A10	107.26	697	3488	6	33	0.186
B01	136.91	1073	3791	8	28	0.084
B02	108.64	710	3580	7	33	0.217
B03	135.60	1427	5133	11	38	0.029
B04	133.21	1047	4276	8	32	0.159
B05	149.44	1011	4433	7	30	0.251
B06	136.72	876	4372	6	32	0.094
B07	125.99	1093	3813	9	30	0.032
B08	129.15	962	3710	7	29	0.074
B09	146.50	654	4409	4	30	0.196
B10	135.81	947	4164	7	31	0.238
A	1025.12	7538	35936	7	35	0.177
B	1337.96	9800	41681	7	31	0.137
AB	2363.08	17338	77617	7	33	0.157

*Chr, Chromosome; r^2^ of chr LD, average LD between all SNPs on a chromosome*.

The selected SNP markers were not evenly distributed across the whole genome, with 7,538 and 9,800 markers on the A and B subgenomes, respectively. Chromosome B03 harbored the highest proportion of SNPs (8.23%; 1,427 of 17,338), while chromosome A08, the shortest chromosome at 48.94 Mb, contained the least (1.81%; 314 of 17,338). The average number of SNPs/Mb was seven on both the A and B subgenomes, while average genes/Mb were 35 and 31, respectively. Chromosome B03 had the highest number of genes (5,188 of 77,617), while its counterpart, chromosome A03, contained the second highest (4,929 of 77,617).

LD was estimated as the *r*^2^ value, revealing uneven distribution of SNPs on each chromosome. *r*^2^ values of the A subgenome ranged from 0.071 on chromosome A03 to 0.356 on chromosome A02, while those of the B subgenome ranged from 0.029 on chromosome B03 to 0.251 on chromosome B05. Average *r*^2^ values of the A and B subgenomes were 0.177 and 0.137, respectively, revealing differences in the level of LD between different chromosomes and subgenomes.

### Population structure and genome-wide divergence in peanut

**Figure 2:**
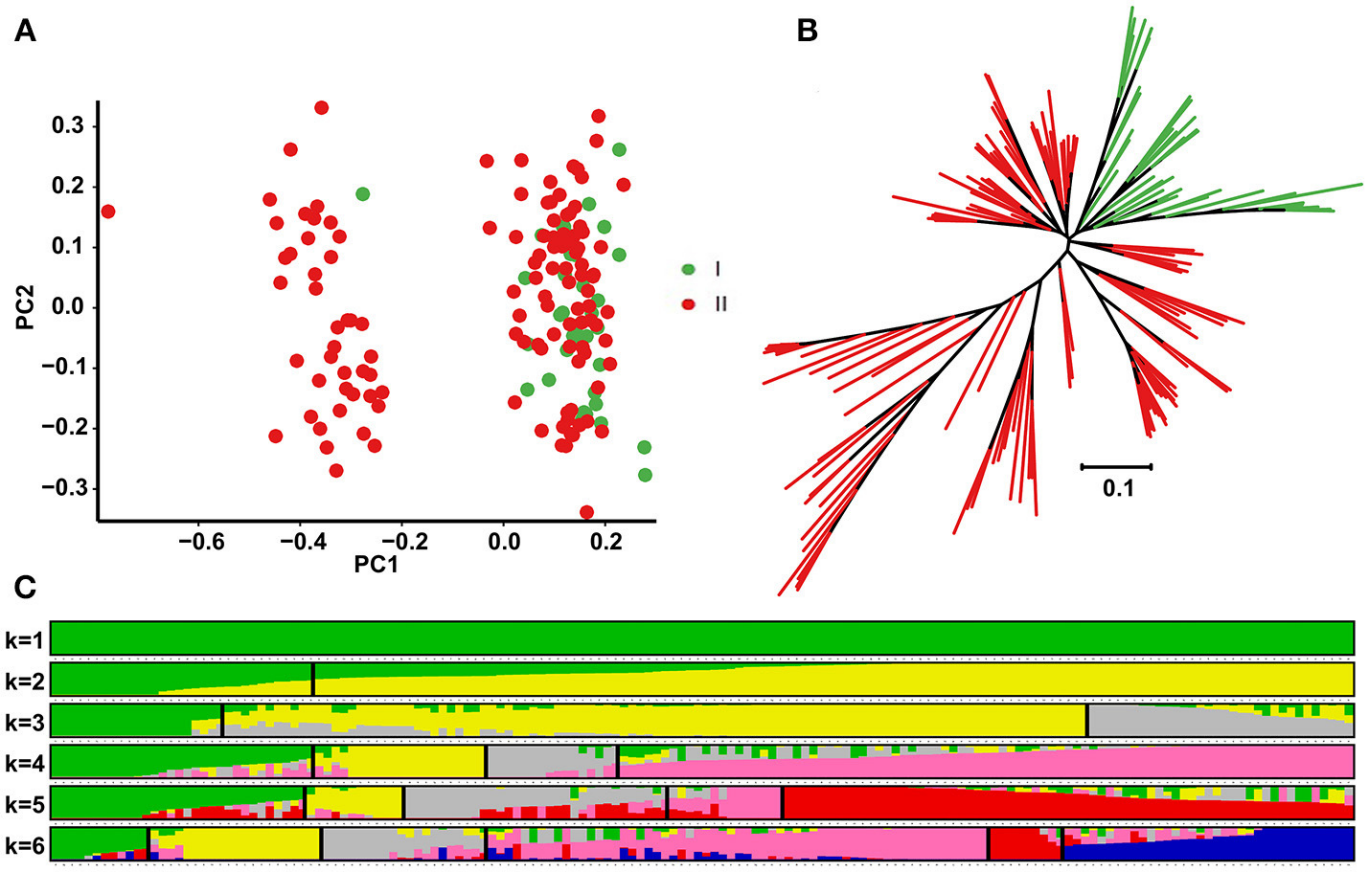
Principal component analysis (PCA), phylogenetic tree construction and population structure analysis of the 158 peanut accessions. **(A)** Scatter plots of the first two principal components. The horizontal and vertical coordinates represent PC1 (with variance explain 8.16%) and PC2 (with variance explain 5.23%). Each dot represents an accession. **(B)** Phylogenetic tree constructed with 17,338 high quality SNPs. **(C)** Population structure dividing the accessions into two groups (ΔK value was 0.47): subsp. *hypogaea* (group I) and subsp. *fastigiata* (group II).

To examine divergence of the 158 accessions during evolution, analysis of population structure, phylogenetic relationships and PCA were carried out using the 17,338 selected SNPs. According to the K genetic clusters, the most likely number of inferred members was 2 with ΔK = 0.47 (Figure 2C). Nevertheless, accessions were classified into three major clusters in the NJ phylogenetic tree, with *hypogaea* accessions (group I) forming a separate cluster, and *fastigiata* accessions (group II) forming another large cluster (Figure 2B). PCA was conducted using the first two principal components (Figure 2A), PC1 (with variance explain 8.16%) and PC2 (with variance explain 5.23%), revealing that the accessions probably divided into two groups, with different degrees of introgression between the two subspecies during cultivation.

### GWAS of loci underlying domestication traits in peanut

**Figure 3:**
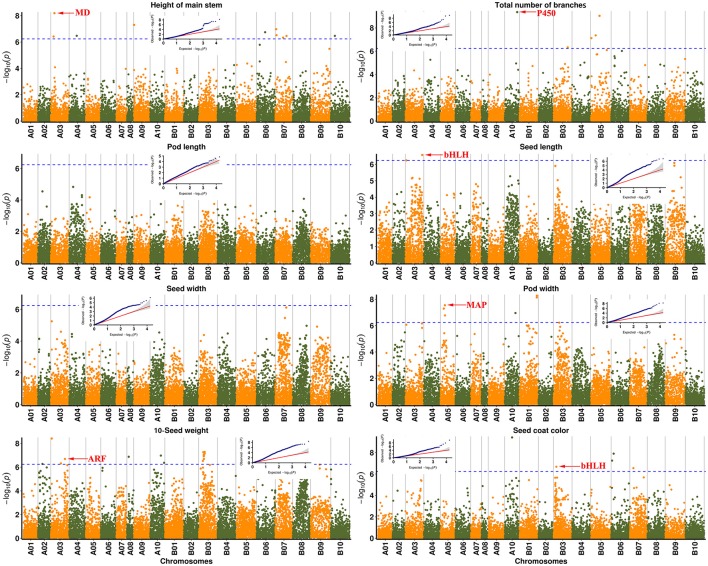
Genome-wide association studies (GWAS) of traits associated with peanut domestication. Manhattan plots with corresponding small QQ plots are shown in each figure of each trait. Associated significant SNPs are marked by arrows with reported candidate genes. The Bonferroni multiple test threshold is shown by a dotted blue line. MD: Malate dehydrogenase; P450: Cytochrome P450 superfamily protein; bHLH: bHLH transcription factor; ARF: Auxin response factor; MAP: Microtubule-associated protein.

Eleven traits were selected for identification of underlying genetic loci and regions. In total, 51 SNP peaks associated with six traits reached the corrected *P* value according to the Bonferroni method (*P* < 5.76e^−07^ at a = 0.01 or −log_10_(P) = 6.238) (Figure 3, Table S3). A 100-kb genomic region on each side of the peak SNP associated with the corresponding traits was subsequently analyzed for identification of candidate genes (Table 2).

**Table 2:** Six significant SNPs and predicted candidate genes associated with major agronomic traits in 158 peanut accessions.

**Trait**	**SNP location (bp)**	**Gene model**	**Distance to SNP (kb)**	**Functional annotation**
HMS	A03-26481539	Aradu 52T5J	32 (26,514,081–26,516,597)	Malate dehydrogenase
TNB	A10-90376017	Aradu J85DC	8 (90,384,547–90,391,660)	Cytochrome P450 superfamily protein
Seed length	A03-6992035	Aradu T1PSR	57 (7,049,674–7,051,562)	bHLH transcription factor
Seed weight				
Seed weight	A03-119879303	Aradu PZ2UH	42 (1,19,921,885–1,19,926,789)	Auxin response factor
Pod weight	A05-32373760	Aradu CVC5Q	32 (32,341,000–32,343,951)	Microtubule-associated protein
Seed coat color	B03-22076736	Araip P4GTD	12 (22,064,568–22,066,022)	bHLH transcription factor

*HMS, Height of main stem; TNB, Total number of branches*.

As a result, a total of 13 significant SNPs were associated with height of the main stem, with the peak SNP A03-26481539 explaining 27.55% of the phenotypic variation. The Aradu 52T5J gene was located ~32 kb from this SNP, and is known to encode a malate dehydrogenase, which is thought to be related to biomass and plant height in maize (Carrari et al., 2005). Peak SNP A10-90376017 for the total number of branches explained 26.64% of the phenotypic variation, and was located only ~8 kb from the nearest gene, Aradu J85DC, which encodes a cytochrome P450 superfamily protein reportedly involved in the strigolactone synthetic pathway in rice (Gomez-Roldan et al., 2008) and soybean. The peak SNP A03-6992035 explained 15.52 and 19.99% of the phenotypic variation of seed length and 10-seed weight, respectively. A candidate gene encoding a bHLH transcription factor was located ~57 kb from this peak, and is thought to be a pleiotropic gene involved in seed development (Kondou et al., 2008). Aradu PZ2UH was located ~42 kb from A03-119879303, another peak SNP related to 10-seed weight (explaining 18.69% phenotypic variation) on chromosome A03, and encodes an auxin response factor (ARF) involved in plant growth and seed development (Okushima et al., 2005; Attia et al., 2009). A peak SNP explaining 26.32% of the phenotypic variation in pod width was located on chromosome A05. One candidate gene, Aradu CVC5Q, was located ~32 kb away, and encodes a microtubule-associated protein (MAP) that reportedly influences seed shape by regulating microtubule growth (Deng et al., 2012). The candidate gene for seed coat color, located ~12 kb from the peak SNP B03-22076736, which explained 21.94% of the phenotypic variation), also represented a bHLH transcription factor previously found to influence seed coat color in rice (Sweeney et al., 2006). These results suggest that GWAS was effective in clarifying candidate genes related to major domestication-related traits in peanut.

### Genomic changes and target regions associated with selection

**Figure 4:**
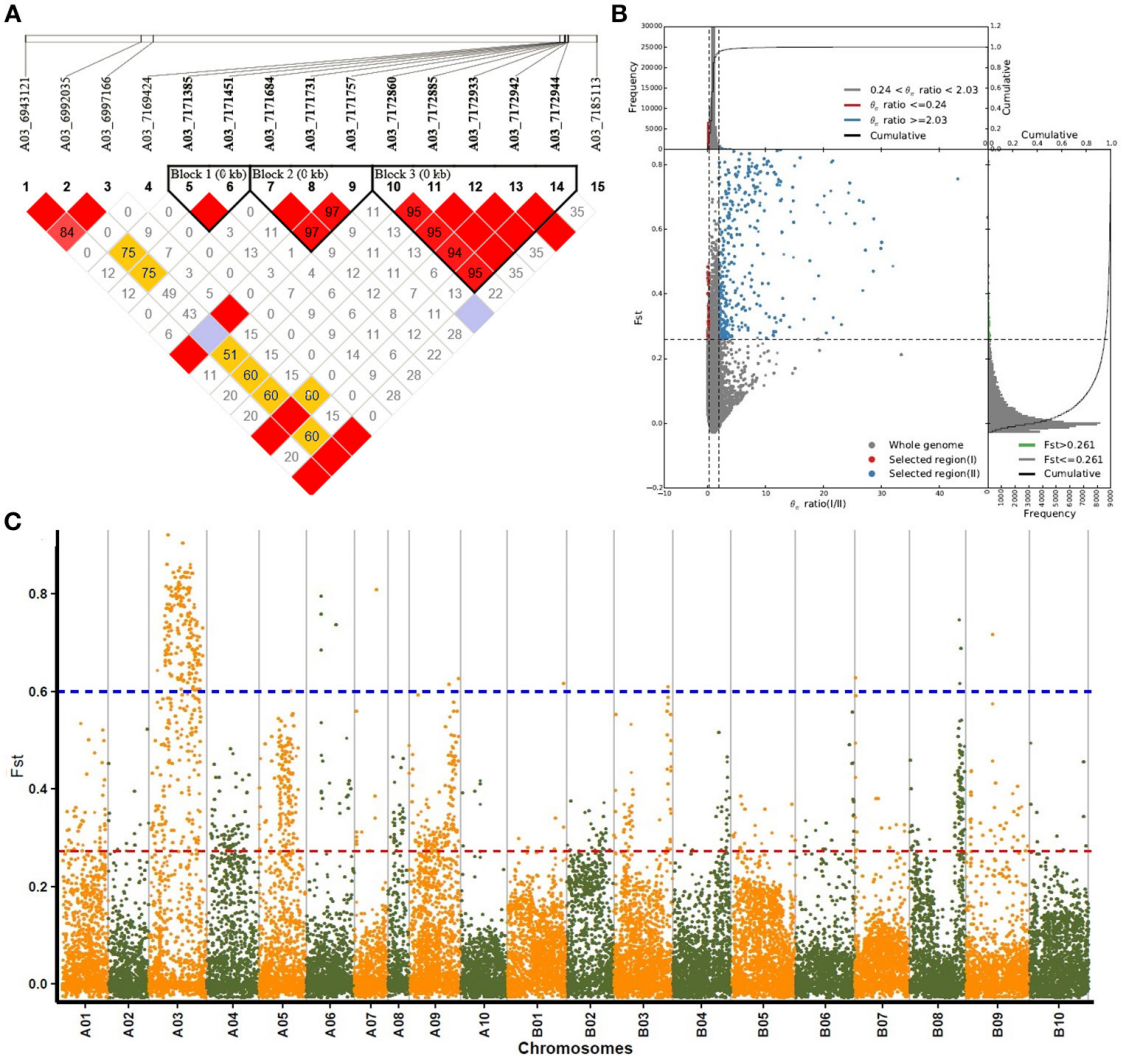
Selective sweeps and population differentiation analyses of subsp. *hypogaea* (group I) and subsp. *fastigiata* (group II). **(A)** Regional plot of 15 SNPs in the 200-kb selective sweep region on chromosome A03. The bottom panel indicates the extent of LD in the region based on pairwise r2-values which are shown in the LD triangles. **(B)** Distributions of selective sweeps in subsp. *hypogaea* (group I) and subsp. *fastigiata* (group II). **(C)** Manhattan plots of FST values of peanut chromosome.

Genomic changes related to selective processes during domestication can be determined using genotypic data. In this study, a total of 1,429 genes were defined in 335 highly differentiated genomic regions in the two groups using an *F*_*ST*_ threshold of 0.261 (determined by the 5% right tails of the *F*_*ST*_ distribution) and a π I / π II ratio threshold of 2.03 (Figures 4B,C). The gene distribution of the resulting sweeps was subsequently determined (Table S4). A03 contained the most number of genes (662 genes, 45.94%), and presented stronger selective sweep signals in group I than group II. A total of 186 and 158 genes were found on chromosomes B03 and B08, respectively, corresponding to 12.91 and 11.06%, respectively, and ranking them second and third during peanut artificial selection. No selective sweep signals were detected on chromosomes B01 or B10.

A total of 15 SNPs were found in the 200-kb selective sweep regions of major peak SNP A03-6992035 (Figure 4A), which is related to seed length and seed weight. Three SNPs were also found in the two gene models, Aradu D69CU and Aradu T1PSR, respectively, both of which encode a bHLH transcription factor. In total, 21 genes were found in this region, and only nine showed annotation. In addition to the two bHLH genes mentioned above, three major intrinsic protein (MIP) genes involved in carbohydrate transport and metabolism, and one F-box gene and one proline-rich protein (PRP) gene both involved in plant growth and stress response, were also identified in this region.

Nucleotide-binding leucine-rich repeat (NB-LRR)-encoding genes are of particular interest because they confer resistance against pests and disease. Possible resistant genes in the highly differentiated genomic regions were therefore analyzed. Ten and 25 genes containing the NB-LRR domains were identified on the A and B subgenomes, respectively. Of the 10 NB-LRR-encoding genes on the A subgenome, eight were located on chromosome A03, and of the 25 on the B subgenome, 18 were located in two genomic regions of chromosome B07. One region was located within 282 kb of chromosome B07 (between 1714315 and 1996823) and harbored 15 NB-LRR-encoding genes (Table S5), suggested that this area contains a resistance gene family. The second region was located between 4775007 and 4820955 on chromosome B07, and included three NB-LRR-encoding genes. Interestingly, five NB-LRR-encoding genes were found in a major selective sweep on chromosome B03. This region covered 6.89 Mb and contained 107 selective genes, including one gene encoding a Gibberellin-related protein and three genes related to flavonoid biogenesis and regulation. These findings suggest that this region plays important roles in both resistance and plant-type-related traits.

## Discussion

Species in the genus *Arachis* are widely distributed across tropical, subtropical and warm temperate zones, but only the cultivated peanut (*A. hypogaea*) is an important food crop. Peanut evolved morphologically during domestication, allowing it to adapt to various agroecological environments (Stalker and Simpson, 1995). Subsp. *fastigiata* has more advanced traits than subsp. *hypogaea* in terms of plant habit and pod morphology, and in this study, represented a larger portion (77.2%) of the selected accessions (Figure 1 and Table S1). In line with this, Krapovickas (1969) postulated that *hypogaea* (subsp. *hypogaea*) represents the most ancient variety due to its runner habit, lack of floral spikes and branching patterns, which are similar to the characteristics of wild *Arachis* species. In this study, analysis of phylogenic relationships, population structure and PCA among 158 peanut accessions revealed that accessions in subsp. *fastigiata* contain unique genomic regions that differ from those in *hypogaea* (Figure 2). Four wild diploid accessions were previously sequenced and removed due to ploidy difference (data not shown). Interestingly, some accessions were not clearly distinguishable, possibly because they underwent differing degrees of genetic introgression during manual selection.

Cultivated peanut has a narrow genetic base, possibly resulting from a single polyploidization event (Kochert et al., 1996), and can therefore be improved using introgression genes for disease resistance and other important agronomic traits from wild species (Dwivedi et al., 2007; Holbrook et al., 2008; Isleib et al., 2011). Molecular markers for economically significant traits have been widely used to improve the speed and efficiency of MAS breeding in peanut (Selvaraj et al., 2009; Chu et al., 2011). SNP markers, the most abundant molecular marker, are a cost-effective method of high-throughput molecular genotyping, but have limited use in peanut because of the homeologous A and B subgenomes. In this study, the two diploid genome (*A. ipaensis* and *A. duranensis*) were used as the reference genomes. In order to accurately allocate marker tags to the correct genome, sequencing depth was combined with integrity and MAF to filter SNPs. There are not too much SNPs found in exceptionally high homologous regions, which would not influence the following GWAS results. Finally, a total of 17,338 high quality SNPs were identified across the whole genome by reduced representation sequencing technology (the SLAF-seq method) (Table 1 and Figure S1). The average sequencing depth was 8.06-fold, and the average number of SNPs / Mb was seven in both the A and B subgenomes, suggesting a reasonable density of markers given the relatively low cost of the genotyping method (He et al., 2011; Li et al., 2013; Morris et al., 2013).

GWAS is considered an efficient method for genetic analysis of complex trait variation (Han et al., 2016; Fahrenkrog et al., 2017). Based on the markers developed in this study, 51 association SNP peaks were identified in this study, along with candidate genes or loci corresponding to domestication-related traits (Figure 3 and Table S3). A total of 13 association peaks for height of the main stem were found on eight chromosomes, while 18 peaks representing seed weight were found on six chromosomes, suggesting that there is a group of QTLs responsible for these traits. The basic transcription factor, bHLH family, is involved in plant growth and developmental processes as well as stress responses and secondary metabolism (Heim et al., 2003), and in this study, was linked with seed shape and seed coat color (Table 2). The Aradu PBR53 gene located ~37 kb from SNP A05-32373760 for pod weight, which was predicted to encode formin protein, a newly revealed regulatory factor of cell skeleton assembly (Guo and Ren, 2006). In *Arabidopsis thaliana*, it was found to play a role in cell division and cell polarity (Zhang et al., 2016), but studies in peanut are limited, making it a good focal point for future research. Using the SNPs identified in this study, further analysis of agronomic traits will be possible, allowing rapid identification of candidate genes for future peanut breeding programs.

Similar to other important crop plants, peanut has undergone continuous selection through domestication and intensive breeding events. Domestication from a wild to a cultivated species is largely associated with genome-wide duplications, mutations, selection and genetic drift (Kim et al., 2010; Li et al., 2013). Many traits thought to be involved in peanut domestication were previously clustered in three genomic regions on chromosomes A07, B02, and B05 (Fonceka et al., 2012), suggesting that several linked genes are responsible for the phenotypic variation in 158 peanut accessions collected from all growing regions of China. In this study, selective sweeps in the two peanut groups were measured using *F*_*ST*_ values and sequence diversity (π) ratios. The existence of major selective sweeps on chromosome A03 indicated that this chromosome was subjected to primary selection pressure (Figure 4). This finding was similar to the results of GWAS, whereby several important genes related to domestication traits were located on chromosome A03. SNPs in these regions are therefore likely to be valuable for MAS breeding.

There are several major diseases, like early leaf spot(caused by *Cercospora arachidicola*), late leaf spot (caused by *Cercosporidium personatum*), and Tomato spotted wilt virus (TSWV, spread by thrips), may cause significant yield loss in peanut production (Nigam et al., 2012). Breeding of resistance cultivars is the most cost-effective method of reducing disease damage in peanuts. The role of NB-LRR proteins in plant defense against pathogens has been extensively studied (DeYoung and Innes, 2006; Nagy and Bennetzen, 2008). In this study, genes encoding NB-LRR protein were mainly distributed on chromosomes A03 and B07, especially in a 280 kb region on B07, which contained 15 promising candidate genes (Table S5). Wang et al. (2013) constructed two genetic maps to identify QTLs for thrips, TSWV and leaf spot (LS) in peanut. One QTL for TSWV, qF_2_TSWV3, was identified in the same marker interval (seq5D5-GM2744) on linkage group AhII with one QTL for LS, qF_2_LS1. Another QTL, qF_5_LS10 for LS, was identified between GM1254 and seq15C10 on linkage LGT17. The two linkage groups, AhII and LGT17, were colinearized with B07 of reference consensus genetic map (the three QTLs mentioned above covered 24 cM), suggested that there is a cluster of resistance QTLs on B07. Pandey et al. (2017) found that of the total 42 QTLs linked to diseases resistance in peanut, 34 were mapped on the A sub-genome and eight mapped on the B sub-genome, suggesting that the A sub-genome harbors more resistance genes than the B sub-genome, which is in agreement with Bertioli et al. (2016), who reported that there are more NB-LRR-encoding disease resistance-like genes in the “A” genome (397 genes in *A. duranensis*) than in the “B” genome (345 genes in *A. ipaensis*). Though function of these genes needs to be further validated, these findings suggest that SNPs located in these major selective sweeps will facilitate future breeding of resistant cultivars in peanut. The major selective sweep on chromosome B03 harbored a number of genes related to both resistance and plant-type-related traits, similar to a recent study (Zhou et al., 2016), suggesting that this genetic region is worthy of further investigation.

## Conclusion

In summary, this study provides the first insight into the complex genetic relationship between agronomic traits and domestication processes in peanut. Chromosomes A03 and B03 harbored major genes related to peanut domestication, while chromosome B07 contained a cluster of NB-LRR-encoding genes. Further studies are now needed to understand the genetic mechanisms underlying yield- and seed-related traits and identify potential resistant genes for future peanut breeding programs.

## Author contributions

XZ, JZ, XH, DY carried out phenotyping and genotyping. XZ, YW, DY managed the project. XZ, XM, DY analyzed the data. XZ wrote the paper. All of the authors read and approved the manuscript.

### Conflict of interest statement

The authors declare that the research was conducted in the absence of any commercial or financial relationships that could be construed as a potential conflict of interest.
