# Supplementary material for: Genome-Wide Association Study of Major Agronomic Traits Related to Domestication in Peanut
Source: Front Plant Sci. 2017 Sep 26;8:1611. doi: 10.3389/fpls.2017.01611 (PMC5623184; doi:10.3389/fpls.2017.01611)
Supplement: Supplementary file 1 [file Table1.DOCX]

Table S1 The phenotype of 11 agronomic traits investigated in 158 peanut accessions used in this study

| Material No. | Name | HMS（cm） | TNB | Pod（cm） | | Seed（cm） | | 10-Pod weight（g） | 10-Seed weight（g） | Seed coat color | Group |
| --- | --- | --- | --- | --- | --- | --- | --- | --- | --- | --- | --- |
|  |  |  |  | Length | Width | Length | Width |  |  |  |  |
| P1 | Huayou6 | 22.00 | 12.00 | 3.00 | 1.47 | 1.67 | 1.27 | 21.63 | 9.40 | Pink | II |
| P10 | Huayou48 | 29.33 | 10.00 | 2.67 | 1.30 | 1.57 | 0.83 | 16.33 | 7.10 | Pink | II |
| P100 | Huayou34 | 35.33 | 9.67 | 3.53 | 1.53 | 2.20 | 1.43 | 33.79 | 13.37 | Pink | II |
| P101 | Huayou71 | 29.67 | 18.33 | 3.60 | 2.10 | 2.23 | 1.13 | 38.33 | 16.10 | Pink | II |
| P102 | Huayou75 | 42.00 | 14.67 | 4.03 | 1.90 | 2.03 | 1.33 | 36.97 | 14.00 | Pink | II |
| P103 | Huayou220 | 43.33 | 7.00 | 3.60 | 2.10 | 1.97 | 1.07 | 36.67 | 15.27 | Pink | II |
| P104 | Huayou221 | 44.00 | 27.00 | 3.73 | 1.73 | 2.23 | 1.13 | 35.33 | 13.53 | Pink | II |
| P105 | Huayou275 | 43.00 | 6.67 | 3.93 | 1.60 | 2.10 | 1.37 | 31.00 | 12.00 | Pink | II |
| P106 | Huayou321 | 38.00 | 9.67 | 4.20 | 1.83 | 2.17 | 1.07 | 38.07 | 15.63 | Pink | II |
| P107 | Hua415 | 33.00 | 27.67 | 5.07 | 1.90 | 2.97 | 1.37 | 46.63 | 19.37 | Pink | II |
| P108 | Hua450 | 31.00 | 9.33 | 3.53 | 1.43 | 2.43 | 1.33 | 36.43 | 15.23 | Purple | I |
| P109 | Hua452 | 32.50 | 10.50 | 2.70 | 1.20 | 2.60 | 1.30 | 27.60 | 12.60 | Purple | I |
| P11 | Huayou51 | 39.00 | 16.33 | 4.73 | 2.10 | 2.40 | 1.47 | 40.93 | 17.33 | Pink | II |
| P110 | Kaibai 2 | 30.00 | 12.67 | 3.90 | 1.27 | 2.40 | 1.10 | 30.63 | 11.83 | White | II |
| P111 | Huayu 33 | 45.00 | 7.00 | 4.47 | 1.87 | 2.33 | 1.33 | 38.41 | 15.10 | Pink | II |
| P112 | Hua0815 | 38.67 | 9.67 | 4.80 | 1.73 | 2.70 | 1.43 | 32.00 | 14.03 | Pink | I |
| P113 | Zhengnonghua 9 | 38.33 | 7.67 | 5.00 | 1.87 | 2.70 | 1.40 | 40.13 | 14.57 | Pink | II |
| P114 | Hua717 | 28.00 | 7.00 | 4.47 | 1.77 | 2.40 | 1.20 | 30.60 | 11.40 | Pink | II |
| P115 | Baisha 1016 | 38.33 | 25.00 | 3.10 | 1.47 | 1.60 | 1.03 | 21.70 | 9.13 | Pink | II |
| P116 | Yuhua 7 | 45.67 | 7.00 | 3.80 | 1.13 | 2.37 | 1.23 | 24.21 | 10.32 | Pink | II |
| P117 | Hua814 | 34.33 | 8.67 | 3.10 | 1.27 | 1.73 | 0.97 | 16.57 | 7.07 | Pink | I |
| P118 | Hua 411 | 46.00 | 11.33 | 4.83 | 1.70 | 2.57 | 1.13 | 30.70 | 12.70 | Pink | I |
| P119 | Shitouqi | 37.00 | 11.67 | 3.53 | 1.50 | 2.23 | 1.33 | 35.18 | 14.40 | Pink | II |
| P12 | Huayou55 | 32.67 | 21.33 | 4.13 | 1.87 | 2.03 | 1.20 | 35.63 | 13.27 | Pink | II |
| P120 | Zhonghua 9 | 32.00 | 31.67 | 3.90 | 1.63 | 2.43 | 1.03 | 24.89 | 9.83 | Black | II |
| P121 | Haihua 1 | 34.33 | 10.33 | 3.80 | 1.50 | 2.10 | 1.20 | 29.63 | 12.00 | Pink | II |
| P122 | Shangtuneryang | 27.00 | 33.00 | 4.10 | 1.47 | 2.23 | 1.03 | 23.80 | 10.20 | Pink | I |
| P123 | Hua 17 | 36.67 | 12.67 | 4.07 | 1.43 | 2.00 | 1.17 | 29.00 | 11.77 | Pink | II |
| P124 | Nongdahua 103 | 33.67 | 16.67 | 3.37 | 1.23 | 2.07 | 1.47 | 28.99 | 11.42 | Pink | II |
| P125 | Shengpuhua 1 | 41.00 | 8.67 | 4.47 | 1.60 | 2.17 | 0.90 | 35.87 | 14.83 | Pink | II |
| P126 | Puhua 9519 | 41.00 | 8.67 | 4.47 | 1.60 | 2.17 | 0.90 | 35.87 | 14.83 | Pink | II |
| P127 | Puhua 28 | 27.33 | 9.67 | 3.37 | 1.37 | 2.27 | 1.13 | 24.63 | 10.30 | Pink | II |
| P128 | Pudonghua 1 | 41.33 | 7.67 | 4.07 | 1.33 | 2.37 | 1.20 | 33.17 | 14.13 | Pink | II |
| P129 | HY661 | 35.00 | 10.00 | 3.30 | 1.40 | 1.67 | 0.91 | 19.83 | 9.10 | Pink | II |
| P13 | Huayou56 | 20.00 | 17.67 | 3.13 | 1.50 | 1.67 | 1.07 | 22.57 | 9.53 | Pink | II |
| P130 | HY961 | 31.00 | 8.00 | 3.03 | 1.40 | 1.57 | 0.90 | 19.73 | 9.00 | Pink | II |
| P131 | MH8 | 38.67 | 41.33 | 3.27 | 0.90 | 1.83 | 1.03 | 17.47 | 6.67 | Purple | I |
| P132 | A,monticila | 15.00 | 50.00 | 1.50 | 0.70 | 1.00 | 0.50 | 10.00 | 4.00 | Pink | I |
| P133 | Huaba7 | 12.00 | 50.00 | 1.50 | 0.70 | 1.00 | 0.50 | 10.00 | 4.00 | Pink | I |
| P134 | Huaba10 | 13.00 | 50.00 | 1.50 | 0.70 | 1.00 | 0.50 | 10.00 | 4.00 | Pink | I |
| P135 | Huaba22 | 12.00 | 50.00 | 1.50 | 0.70 | 1.00 | 0.50 | 10.00 | 4.00 | Pink | II |
| P136 | Hua1046 | 40.00 | 9.00 | 3.53 | 1.80 | 1.77 | 0.93 | 26.77 | 10.43 | Pink | II |
| P137 | Hua1047 | 40.00 | 9.00 | 3.53 | 1.80 | 1.77 | 0.93 | 26.77 | 10.43 | Pink | II |
| P138 | Hua1059 | 40.00 | 9.00 | 3.53 | 1.53 | 2.20 | 1.43 | 33.79 | 13.37 | Pink | II |
| P139 | Hua1060 | 40.00 | 9.00 | 3.53 | 1.53 | 2.20 | 1.43 | 33.79 | 13.37 | Pink | II |
| P14 | Hua4098 | 40.89 | 10.33 | 4.86 | 1.81 | 2.33 | 1.29 | 36.90 | 14.41 | Pink | II |
| P140 | Hua2010 | 40.00 | 9.00 | 3.53 | 1.53 | 2.20 | 1.43 | 33.79 | 13.37 | Pink | II |
| P141 | Hua1052 | 40.00 | 9.00 | 3.53 | 1.53 | 2.20 | 1.43 | 33.79 | 13.37 | Pink | II |
| P142 | Hua2011 | 40.00 | 9.00 | 3.53 | 1.53 | 2.20 | 1.43 | 33.79 | 13.37 | Pink | II |
| P143 | Hua2012 | 40.00 | 9.00 | 3.53 | 1.53 | 2.20 | 1.43 | 33.79 | 13.37 | Pink | II |
| P144 | Hua2013 | 40.00 | 9.00 | 3.53 | 1.80 | 1.77 | 0.93 | 26.77 | 10.43 | Pink | II |
| P145 | Hua2014 | 40.00 | 9.00 | 3.53 | 1.80 | 1.77 | 0.93 | 26.77 | 10.43 | Pink | II |
| P146 | Hua2015 | 40.00 | 9.00 | 3.53 | 1.80 | 1.77 | 0.93 | 26.77 | 10.43 | Pink | II |
| P147 | Hua2016 | 40.00 | 9.00 | 4.73 | 2.10 | 2.40 | 1.47 | 40.93 | 17.33 | Pink | II |
| P148 | Hua2017 | 40.00 | 9.00 | 4.73 | 2.10 | 2.40 | 1.47 | 40.93 | 17.33 | Pink | II |
| P149 | Hua2018 | 40.00 | 9.00 | 3.53 | 1.53 | 2.20 | 1.43 | 33.79 | 13.37 | Pink | II |
| P15 | D57 | 36.33 | 12.00 | 2.23 | 0.97 | 1.60 | 1.03 | 14.86 | 5.51 | Pink | II |
| P150 | Huayu 963 | 40.00 | 9.00 | 3.53 | 1.53 | 2.20 | 1.43 | 33.79 | 13.37 | Pink | II |
| P151 | Wh554 | 87.33 | 9.33 | 4.00 | 1.47 | 1.50 | 0.77 | 21.30 | 4.57 | Pink | I |
| P152 | Zhonghua 16 | 30.00 | 8.33 | 3.93 | 1.23 | 2.23 | 1.23 | 28.25 | 10.91 | Pink | II |
| P154 | Wh5633 | 36.00 | 11.67 | 3.70 | 1.63 | 2.70 | 1.10 | 31.78 | 14.34 | Pink | II |
| P155 | ZH0109 | 38.50 | 17.50 | 2.95 | 1.35 | 1.55 | 1.00 | 20.20 | 7.75 | Pink | II |
| P156 | ZH4434 | 41.33 | 12.67 | 3.50 | 1.33 | 2.17 | 1.13 | 25.60 | 8.96 | Pink | II |
| P157 | Zh.h4434 | 31.00 | 13.00 | 3.55 | 1.65 | 1.80 | 1.05 | 23.20 | 9.50 | Purple | II |
| P158 | Hua1321 | 50.00 | 10.00 | 4.57 | 1.57 | 2.47 | 1.40 | 32.67 | 14.67 | Black | II |
| P159 | Hua1322 | 40.00 | 10.00 | 4.20 | 1.63 | 2.00 | 1.33 | 31.33 | 13.10 | Black | II |
| P16 | Huayou76 | 33.33 | 18.67 | 3.37 | 1.70 | 1.83 | 1.37 | 23.57 | 10.23 | Pink | II |
| P160 | Hua1324 | 50.00 | 10.00 | 4.57 | 1.57 | 2.47 | 1.40 | 32.67 | 14.67 | Pink | II |
| P17 | Huayou117 | 41.00 | 11.67 | 4.07 | 1.93 | 2.20 | 1.23 | 29.10 | 12.03 | Pink | II |
| P18 | Huayou123 | 42.33 | 14.33 | 3.43 | 1.63 | 1.80 | 1.07 | 25.27 | 9.77 | Pink | II |
| P19 | Huayou176 | 36.67 | 7.00 | 4.57 | 1.57 | 2.47 | 1.40 | 32.67 | 14.67 | Pink | II |
| P2 | Huayou12 | 26.33 | 19.00 | 3.00 | 1.47 | 1.67 | 1.27 | 21.63 | 9.40 | Pink | II |
| P20 | Huayou181 | 41.00 | 9.50 | 4.65 | 1.95 | 2.50 | 1.45 | 37.15 | 16.30 | Pink | II |
| P21 | Huayou182 | 40.00 | 12.00 | 3.47 | 1.07 | 2.23 | 1.10 | 22.02 | 9.44 | Pink | II |
| P22 | Huayou185 | 26.00 | 9.00 | 3.43 | 1.40 | 1.90 | 1.13 | 19.90 | 8.10 | Pink | II |
| P23 | Huayou193 | 41.67 | 7.67 | 4.20 | 1.63 | 2.00 | 1.33 | 31.33 | 13.10 | Pink | II |
| P24 | Huayou197 | 42.33 | 31.33 | 4.10 | 1.30 | 2.50 | 1.53 | 46.83 | 16.94 | Pink | II |
| P25 | Huayou222 | 40.00 | 10.00 | 3.53 | 1.80 | 1.77 | 0.93 | 26.77 | 10.43 | Pink | II |
| P26 | Huayou268 | 36.00 | 8.00 | 4.17 | 1.43 | 2.57 | 1.33 | 37.23 | 15.76 | Pink | II |
| P27 | Huayou269 | 32.67 | 6.67 | 3.33 | 1.10 | 2.10 | 1.10 | 24.23 | 10.97 | Pink | II |
| P28 | Huayou271 | 40.00 | 7.00 | 3.07 | 1.37 | 1.57 | 0.87 | 18.77 | 7.87 | Pink | II |
| P29 | Huayou294 | 44.00 | 8.00 | 3.90 | 1.13 | 2.37 | 1.27 | 27.68 | 10.98 | Pink | II |
| P3 | Huayou15 | 27.33 | 13.00 | 3.07 | 1.53 | 1.57 | 1.00 | 22.33 | 9.77 | Pink | II |
| P30 | Huayou299 | 43.00 | 21.33 | 4.97 | 1.93 | 2.47 | 1.27 | 40.93 | 16.90 | Pink | II |
| P31 | Huayou313 | 35.67 | 6.67 | 4.17 | 2.10 | 2.13 | 1.20 | 34.53 | 11.57 | Pink | I |
| P32 | Huayou357 | 48.67 | 8.00 | 4.50 | 1.90 | 2.13 | 0.93 | 42.77 | 17.23 | Pink | II |
| P34 | Huayou377 | 36.67 | 9.00 | 5.00 | 1.70 | 2.90 | 1.50 | 43.70 | 15.17 | Purple | I |
| P35 | Huayou445 | 35.33 | 5.33 | 4.30 | 1.70 | 3.07 | 1.73 | 42.80 | 20.80 | Purple | I |
| P36 | Huayou448 | 34.67 | 8.00 | 4.30 | 2.33 | 2.70 | 1.70 | 43.97 | 20.10 | Purple | I |
| P37 | Huayou449 | 35.33 | 5.00 | 3.67 | 1.50 | 2.13 | 1.27 | 31.13 | 14.13 | Purple | I |
| P38 | Xingjiman | 37.00 | 13.00 | 4.00 | 1.73 | 2.00 | 1.03 | 24.43 | 9.20 | Pink | I |
| P39 | Suhei2 | 38.33 | 16.33 | 4.10 | 1.17 | 2.30 | 1.10 | 25.96 | 8.90 | Pink | II |
| P4 | Kainong 71 | 37.33 | 8.67 | 3.70 | 1.57 | 1.97 | 1.13 | 24.47 | 11.13 | Pink | I |
| P40 | Yuhua 36 | 35.33 | 7.67 | 4.03 | 1.60 | 2.27 | 1.13 | 26.23 | 11.03 | Pink | II |
| P41 | Yuhua 37 | 49.67 | 8.33 | 3.63 | 1.50 | 2.37 | 1.30 | 32.43 | 13.21 | Pink | I |
| P42 | Yuhua 9327 | 36.67 | 9.33 | 3.27 | 1.40 | 1.80 | 1.07 | 21.80 | 8.17 | Pink | II |
| P43 | Pudonghua 1 | 33.00 | 9.75 | 4.73 | 1.83 | 2.33 | 1.30 | 37.95 | 14.35 | Pink | II |
| P44 | Zhengnonghua 7 | 44.67 | 7.67 | 4.67 | 1.83 | 2.30 | 1.30 | 35.83 | 14.37 | Pink | I |
| P45 | Shiyouhong 4 | 51.67 | 6.67 | 4.57 | 1.77 | 2.47 | 1.33 | 37.33 | 15.33 | Pink | II |
| P46 | Huayu 26 | 41.67 | 5.67 | 4.47 | 1.80 | 2.33 | 1.27 | 28.57 | 12.10 | Purple | II |
| P47 | Huayu 50 | 35.67 | 10.00 | 4.43 | 1.70 | 2.53 | 1.33 | 37.83 | 16.27 | Pink | II |
| P48 | Huayu50 | 47.00 | 12.67 | 4.40 | 1.50 | 2.30 | 1.20 | 34.27 | 12.97 | Pink | I |
| P49 | Japanxiangxiang | 40.67 | 8.33 | 4.03 | 1.47 | 2.27 | 1.23 | 32.43 | 13.70 | Pink | II |
| P5 | Huayou20 | 36.67 | 15.33 | 3.33 | 1.53 | 1.70 | 1.17 | 21.47 | 9.17 | Pink | II |
| P50 | Tianfu 28 | 48.67 | 6.67 | 2.53 | 0.93 | 1.93 | 0.93 | 16.33 | 6.20 | Black | II |
| P51 | Zhongkaihua 44 | 34.67 | 8.33 | 4.83 | 1.73 | 2.40 | 1.37 | 33.00 | 13.27 | Pink | II |
| P52 | he bei gao you | 36.67 | 9.67 | 3.30 | 1.43 | 2.07 | 1.00 | 18.67 | 6.90 | Pink | II |
| P53 | Yueyou 45 | 42.33 | 10.00 | 4.00 | 1.73 | 2.13 | 1.50 | 33.27 | 10.90 | Pink | II |
| P54 | Jihua 4 | 38.33 | 13.00 | 3.80 | 1.10 | 2.40 | 1.10 | 21.23 | 8.50 | Pink | II |
| P55 | Jihua 10 | 41.00 | 19.33 | 3.70 | 1.53 | 2.07 | 1.10 | 23.30 | 10.83 | Pink | I |
| P56 | Hua0817 | 49.67 | 6.67 | 4.50 | 1.97 | 2.27 | 1.10 | 45.10 | 13.10 | Pink | II |
| P57 | Shuangdun 1 | 41.67 | 13.00 | 4.03 | 1.73 | 2.20 | 1.23 | 38.37 | 13.70 | Pink | I |
| P58 | Hua689 | 30.00 | 5.67 | 4.37 | 1.67 | 2.83 | 1.37 | 35.77 | 16.30 | Pink | II |
| P59 | Yuhua 15 | 35.50 | 12.00 | 3.70 | 1.30 | 2.25 | 1.15 | 14.09 | 11.16 | Pink | II |
| P6 | Huayou30 | 35.67 | 26.67 | 3.53 | 1.80 | 1.77 | 0.93 | 26.77 | 10.43 | Pink | II |
| P60 | Puhua33 | 35.00 | 7.00 | 4.47 | 1.70 | 2.40 | 1.30 | 33.00 | 13.67 | Pink | I |
| P61 | Pukehua 15 | 41.67 | 8.67 | 4.00 | 1.37 | 2.57 | 1.07 | 27.53 | 10.99 | Pink | II |
| P62 | Hua2 | 24.00 | 6.50 | 4.55 | 1.80 | 2.55 | 1.50 | 47.50 | 19.65 | Pink | II |
| P63 | Haihua 2 | 27.00 | 18.00 | 4.03 | 1.67 | 2.20 | 1.10 | 33.13 | 12.43 | Pink | II |
| P64 | Hua U606 | 51.67 | 8.67 | 4.07 | 1.63 | 2.10 | 1.17 | 31.93 | 12.20 | Pink | II |
| P65 | Luhua 11 | 42.00 | 8.33 | 4.10 | 1.80 | 2.27 | 1.30 | 29.63 | 12.10 | Pink | II |
| P66 | Huayou96 | 39.67 | 10.00 | 2.93 | 1.33 | 1.53 | 1.03 | 18.40 | 8.00 | Pink | II |
| P67 | Huayou234 | 50.00 | 7.00 | 5.90 | 2.53 | 3.30 | 1.83 | 70.33 | 25.03 | Pink | II |
| P68 | Hua0814 | 36.67 | 5.67 | 4.40 | 1.87 | 2.43 | 1.07 | 38.50 | 16.20 | Pink | I |
| P69 | Hua0816 | 43.67 | 9.33 | 4.33 | 1.50 | 2.70 | 1.17 | 29.90 | 14.23 | Pink | II |
| P7 | Huayou314 | 38.00 | 29.33 | 3.80 | 1.43 | 2.50 | 1.53 | 27.33 | 12.63 | Pink | II |
| P70 | Hua 410 | 34.33 | 9.33 | 3.67 | 1.53 | 2.07 | 1.13 | 24.33 | 12.67 | Pink | II |
| P71 | Hua 416 | 35.50 | 8.00 | 3.33 | 1.43 | 2.10 | 1.13 | 20.10 | 8.47 | Pink | II |
| P72 | Yuanza 9102 | 37.00 | 12.00 | 3.20 | 1.60 | 1.70 | 1.20 | 26.20 | 11.70 | Pink | II |
| P73 | Huayou216 | 40.33 | 20.67 | 3.90 | 1.80 | 1.97 | 1.33 | 29.83 | 12.93 | Pink | II |
| P74 | Huayou225 | 38.00 | 14.33 | 3.83 | 1.23 | 2.33 | 1.23 | 32.67 | 12.81 | Pink | II |
| P75 | Huayou238 | 47.00 | 15.00 | 4.20 | 1.73 | 2.37 | 1.27 | 32.70 | 12.10 | Pink | II |
| P76 | Huayou252 | 34.67 | 10.00 | 3.53 | 1.37 | 1.93 | 1.13 | 19.47 | 8.67 | Pink | II |
| P77 | Huayou253 | 38.00 | 7.67 | 3.37 | 1.37 | 1.73 | 1.03 | 18.77 | 8.47 | Pink | II |
| P78 | Huayou255 | 33.00 | 6.33 | 3.63 | 1.37 | 1.93 | 1.13 | 21.83 | 9.23 | Pink | II |
| P79 | Huayou26 | 38.00 | 18.00 | 3.53 | 1.80 | 1.77 | 0.93 | 26.77 | 10.43 | Pink | II |
| P8 | Huayou2 | 47.67 | 16.00 | 4.30 | 1.57 | 2.37 | 1.07 | 25.23 | 9.63 | Pink | II |
| P80 | Hua814 | 40.33 | 6.67 | 3.07 | 1.10 | 1.77 | 1.17 | 16.10 | 6.27 | Pink | I |
| P81 | D56 | 57.33 | 4.33 | 3.33 | 1.50 | 1.53 | 0.87 | 23.00 | 4.77 | Pink | I |
| P82 | D79 | 40.33 | 17.33 | 3.37 | 1.03 | 2.33 | 1.10 | 27.47 | 9.51 | Pink | I |
| P83 | ICGSGE18 | 41.00 | 10.00 | 3.23 | 1.03 | 1.87 | 0.87 | 17.02 | 6.85 | Pink | II |
| P84 | WH1506 | 72.33 | 7.00 | 3.80 | 1.60 | 2.23 | 1.33 | 16.10 | 6.47 | Purple | II |
| P85 | WH3211 | 49.33 | 5.33 | 4.27 | 1.43 | 2.00 | 0.97 | 21.90 | 6.80 | Purple | I |
| P86 | WH3646 | 44.00 | 14.33 | 2.37 | 1.07 | 1.50 | 0.90 | 13.33 | 5.24 | Pink | II |
| P87 | WH4230 | 53.67 | 28.00 | 3.33 | 1.33 | 1.97 | 0.80 | 21.00 | 7.40 | Pink | I |
| P88 | WH4307 | 57.00 | 13.33 | 2.60 | 1.20 | 1.50 | 1.03 | 12.53 | 5.60 | Pink | II |
| P89 | Baisha 1017 | 31.33 | 8.33 | 2.90 | 1.63 | 1.60 | 1.13 | 20.63 | 8.03 | Pink | II |
| P9 | Huayou45 | 33.00 | 11.33 | 3.77 | 1.70 | 2.10 | 1.20 | 28.73 | 11.22 | Pink | II |
| P90 | Fuhuasheng | 38.00 | 12.00 | 3.40 | 1.53 | 1.83 | 1.07 | 23.80 | 9.47 | Pink | I |
| P91 | Huagaoyou | 36.33 | 22.00 | 2.70 | 1.40 | 1.63 | 1.07 | 16.20 | 6.77 | Pink | I |
| P92 | hua pi | 58.33 | 13.67 | 2.70 | 1.03 | 1.70 | 1.00 | 17.27 | 6.02 | Purple | I |
| P93 | Huayu 34 | 42.33 | 8.33 | 3.30 | 1.47 | 1.50 | 0.83 | 19.37 | 7.70 | Pink | I |
| P94 | Huayu 51 | 31.00 | 10.33 | 3.33 | 1.47 | 2.43 | 1.53 | 20.83 | 8.95 | Pink | I |
| P95 | Hengchuanzhigan | 40.67 | 11.67 | 4.07 | 1.70 | 2.30 | 1.27 | 32.33 | 12.63 | Pink | I |
| P96 | Kaixuan 08 | 31.33 | 15.33 | 2.77 | 1.23 | 1.67 | 1.10 | 15.73 | 7.47 | Pink | II |
| P97 | Tianfu 22 | 31.67 | 10.33 | 2.90 | 1.17 | 1.73 | 0.93 | 16.86 | 7.23 | Pink | II |
| P98 | Tianhuasheng | 38.67 | 15.67 | 4.67 | 1.70 | 2.23 | 1.17 | 34.47 | 13.17 | Purple | II |
| P99 | Huayou7 | 26.33 | 8.33 | 2.60 | 1.70 | 1.70 | 1.00 | 10.64 | 5.07 | Pink | II |

Note: Each value is the average of three independent biological replicates. The accession is grouped as I and II according to the agronomic traits. HMS: Height of main stem; TNB: Total number of branches.
