# Supplementary material for: Genome-Wide Association Study of Major Agronomic Traits Related to Domestication in Peanut
Source: Front Plant Sci. 2017 Sep 26;8:1611. doi: 10.3389/fpls.2017.01611 (PMC5623184; doi:10.3389/fpls.2017.01611)
Supplement: Supplementary file 2 [file Table2.DOCX]

Table S2 The descriptive statistics of phenotypic variation for eight traits in 158 peanut accessions

| Trait | Min | Max | Mean±SE | SD | CV(%) |
| --- | --- | --- | --- | --- | --- |
| Height of main stem (cm) | 12.0 | 87.3 | 38.3±0.72 | 9.06 | 23.65 |
| Total number of branches | 4.3 | 50.0 | 12.8±0.68 | 8.57 | 66.98 |
| Pod length (cm) | 1.5 | 5.9 | 3.7±0.06 | 0.72 | 19.35 |
| Pod width (cm) | 0.7 | 2.5 | 1.5±0.02 | 0.31 | 19.93 |
| Seed length (cm) | 1.0 | 3.3 | 2.1±0.03 | 0.39 | 18.63 |
| Seed width (cm) | 0.5 | 1.8 | 1.2±0.02 | 0.22 | 19.20 |
| Pod weight (g) | 10.0 | 70.3 | 28.5±0.73 | 9.10 | 31.98 |
| Seed weight (g) | 4.0 | 25.0 | 11.5±0.29 | 3.68 | 32.01 |

Note:

Min: Minimum; Max: Maximum; SE: Standard Error; SD: Standard Deviation; CV: Coefficient of Variation.
