# Supplementary material for: Genome-Wide Association Study of Major Agronomic Traits Related to Domestication in Peanut
Source: Front Plant Sci. 2017 Sep 26;8:1611. doi: 10.3389/fpls.2017.01611 (PMC5623184; doi:10.3389/fpls.2017.01611)
Supplement: Supplementary file 4 [file Table4.DOC]

Table S4 Summary of the 1429 genes in major selective sweep regions detected on chromosome

of the 158 peanut accessions

| Chr | Region* | Genes | Chr | Region | Genes |
| --- | --- | --- | --- | --- | --- |
| A01 | 54 | 71 | B01 | - | - |
| A02 | 26 | 7 | B02 | 18 | 13 |
| A03 | 1267 | 662 | B03 | 113 | 186 |
| A04 | 147 | 49 | B04 | 10 | 12 |
| A05 | 266 | 61 | B05 | 7 | 5 |
| A06 | 58 | 23 | B06 | 31 | 23 |
| A07 | 23 | 12 | B07 | 62 | 91 |
| A08 | 20 | 3 | B08 | 157 | 158 |
| A09 | 31 | 28 | B09 | 65 | 22 |
| A10 | 12 | 3 | B10 | - | - |
| Total | 1904 | 919 | Total | 463 | 510 |

Note: The reference genomes were *A.duranensis* (AA) and *A.ipaensis* (BB) in this study.

*: Region was determined by the intersection of the top 5% of the *F_ST_*values and the top 5% of the π I / π II ratios.
