# Supplementary material for: Genome-Wide Association Study of Major Agronomic Traits Related to Domestication in Peanut
Source: Front Plant Sci. 2017 Sep 26;8:1611. doi: 10.3389/fpls.2017.01611 (PMC5623184; doi:10.3389/fpls.2017.01611)
Supplement: Supplementary file 5 [file Table5.DOCX]

Table S5 The gene model and position of 15 genes located on chromosome B07 of peanut

Note: Reference genome was *A. ipaensis.*

| Gene model | Position |
| --- | --- |
| Araip XA728 | 1714315-1717530 |
| Araip GS257 | 1718459-1754487 |
| Araip GWC3K | 1754549-1757341 |
| Araip DBF4A | 1760855-1761208 |
| Araip K3EAN | 1761266-1775540 |
| Araip 3M3GN | 1790338-1801369 |
| Araip 9EM51 | 1801413-1813246 |
| Araip 1EG9W | 1826907-1836058 |
| Araip EE6SM | 1837405-1838310 |
| Araip 5SL6S | 1838387-1839943 |
| Araip W1G8F | 1869761-1872544 |
| Araip L1T3E | 1880809-1883555 |
| Araip IG4AH | 1884416-1887568 |
| Araip FZ6IJ | 1983896-1985085 |
| Araip 35N47 | 1993846-1996823 |
