# Supplementary material for: Genome-Wide Association Study of Major Agronomic Traits Related to Domestication in Peanut
Source: Front Plant Sci. 2017 Sep 26;8:1611. doi: 10.3389/fpls.2017.01611 (PMC5623184; doi:10.3389/fpls.2017.01611)
Supplement: Supplementary file 6 [file Image1.PDF]

Fig S1 Distribution of 17,338 polymorphic SNPs on 20 chromosomes of peanut based on 158 accessions

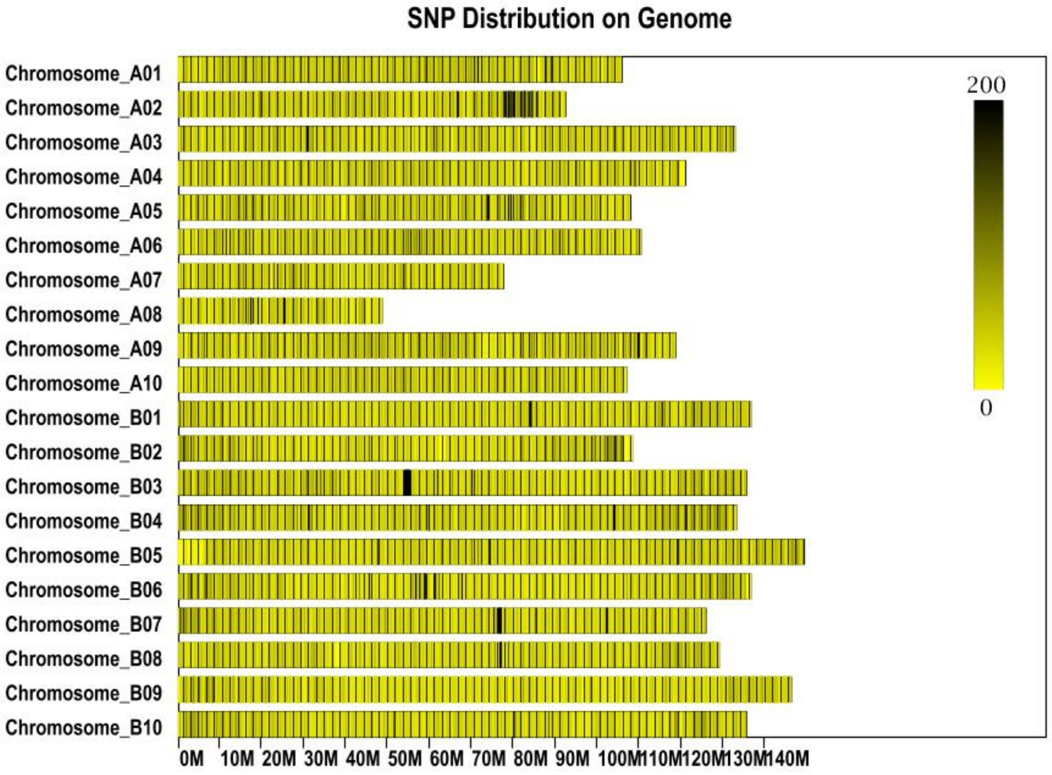

Note: The yellow bar denotes an chromosome with length. The genome is divided according to the window size of 1M, means that the more SNPs in each window, the darker the color is. Area with deeper color is the area in which the SNPs is concentrated. The reference genomes were *A. duranensis* (AA) and A. *ipaensis* (BB).
